# Multi-Feature Fusion and Optimization for *Micropterus salmoides* Tracking and Body Length Monitoring in Complex Aquaculture Environments

**DOI:** 10.3390/s26134250

**Published:** 2026-07-04

**Authors:** Ziyi Yin, Guanxu Li, Zhiyi Liu, Feng Liu, Mai Li, Chengguo Wang

**Affiliations:** 1 Yantai Institute, China Agricultural University, Yantai 264670, China; yinziyi@cau.edu.cn (Z.Y.); liguanxu@cau.edu.cn (G.L.); 13281266485@163.com (Z.L.); liufeng2008@cau.edu.cn (F.L.); 2 Shandong Key Laboratory of Digital Fishery, Yantai 264670, China; 3 Institute of Agricultural Engineering, Chongqing Academy of Agricultural Sciences, Chongqing 401329, China; rlm410@163.com

**Keywords:** YOLOv8, *Micropterus salmoides*, computer vision, target detection, multi-target tracking

## Abstract

To achieve non-contact and continuous monitoring of body length in *Micropterus salmoides* and overcome the stress damage and subjective error associated with traditional manual measurement, this paper proposes an improved YOLOv8-based multi-target tracking framework for intensive recirculating aquaculture systems. The system employs a geometric measurement framework based on monocular vision that achieves conversion from pixel coordinates to actual body length through camera calibration, water-surface refraction correction, and pose projection correction. Under a collaborative optimization framework integrating detection and tracking, the model incorporates multi-scale feature enhancement, lightweight re-identification (ReID), and a robust data association mechanism, which improves system stability under conditions of high fish density, variable illumination, and turbid water. A shallow feature fusion path is introduced to enhance small-target perception, and a MobileNetV3_ReID model is adopted to extract highly discriminative appearance features, which improves identity consistency while maintaining model compactness. In the data association stage, a hybrid cost matrix integrating IoU, cosine similarity, and motion consistency is constructed, and optimal matching is realized through the Hungarian algorithm. Dynamic threshold adjustment and an exponential moving-average feature-update strategy are introduced to effectively suppress identity switching. Experiments were conducted on an overhead video dataset of *Micropterus salmoides* collected at a recirculating aquaculture system facility. The results show that the proposed method achieves 82.7% mAP50 while maintaining a real-time throughput of 88 FPS, with MOTA reaching 76.9% and IDF1 reaching 81.5%—the latter representing an improvement of 3.2 percentage points over BoT-SORT and 5.3 percentage points over the YOLOv8 baseline tracker. The number of identity switches (IDSW) decreased from 89 in the baseline configuration to 39, a reduction of 56.2%. Crucially, these component-level improvements translate into a body length error (BLE) of 5.2 ± 1.8% (MAE = 1.35 cm, Pearson r = 0.972), representing a 38.8% improvement over the baseline BLE of 8.5% and satisfying the 5–10% tolerance required for aquaculture growth monitoring. Ablation analysis confirms that both detection enhancements (contributing −1.3% BLE) and tracking optimizations (contributing −2.0% BLE) are necessary to achieve this application-level accuracy.

## 1. Introduction

*Micropterus salmoides* is an important freshwater aquaculture species in China. According to statistics from the Ministry of Agriculture and Rural Affairs of China (2025), the total output of aquatic products in China in the first half of 2025 reached 33.6453 million tons, an increase of 4.59% year-on-year. Among these, the total output of *Micropterus salmoides* aquaculture exceeded 800,000 tons, approaching that of grass carp (*Ctenopharyngodon idella*), one of the most commonly consumed fish in China. As the highest-yielding single species in Chinese aquaculture, *M. salmoides* is sometimes referred to as the “fifth major domesticated fish.” With the increasing intensification of *M. salmoides* farming, the need for accurate assessment of growth status has become increasingly urgent. Body length, as a core indicator for evaluating the growth and development of *M. salmoides*, directly influences feeding strategy, hierarchical management decisions, and harvest timing, and is a key parameter for achieving refined aquaculture management.

Traditional body length measurement of *M. salmoides* relies on manual operation. Fish must be captured, removed from the water, and immobilized before measurement with a ruler or calipers. This process causes significant stress, increases the risk of injury and mortality, and is time-consuming, labor-intensive, and inefficient. Due to operational costs and manpower constraints, the frequency of routine monitoring is typically low, and the number of individuals sampled in each round is limited, making it difficult to achieve continuous tracking and comprehensive evaluation of population growth trends. In addition, measurement results are susceptible to subjective judgment by operators, and the lack of standardized data-recording practices further limits the comparability and analytical value of the data. Therefore, the development of automatic, non-contact, non-invasive, and long-term stable body length monitoring methods represents an important direction for promoting the intelligent upgrading of aquaculture.

Non-contact body length measurement technology provides a viable solution to these challenges. Among available approaches, vision-based estimation algorithms have attracted significant attention owing to their high efficiency and non-destructive nature. Early semi-automatic schemes still relied on manual intervention, offering only limited efficiency improvements. For instance, a semi-automatic measurement system utilizing template matching, developed by Shafait et al. [1], achieved a sixfold increase in measurement speed with an error of approximately 1%, albeit requiring manual initialization and correction, which limits its application in high-throughput, unattended aquaculture monitoring. Advances in deep learning have facilitated accurate localization of multiple individuals and extraction of contour information from single-frame images. Consequently, neural network-based object detection and semantic segmentation have become the mainstream approaches for fish size and length estimation. The IMAFISH_ML system, developed by Navarro et al. [2], employed traditional image-processing algorithms to automatically measure 27 morphological characteristics of fish species such as gilthead seabream (*Sparus aurata*). However, this process requires ex situ imaging on a specific background plate, which significantly diverges from real-world aquaculture conditions. To achieve non-destructive monitoring of free-swimming fish, recent studies have focused on overcoming environmental disturbances in aquaculture imaging. Salman et al. [3] pioneered the application of CNNs for unconstrained underwater fish species classification, demonstrating the ability of CNNs to automatically learn features robust to illumination and background variations, significantly outperforming traditional hand-crafted feature methods and laying a crucial foundation for subsequent deep learning applications in complex tasks such as body length estimation. To address imaging distortions at the water–air interface, Al-Jubouri et al. [4] proposed a comprehensive mathematical model incorporating background subtraction, morphological filtering, and physical optics-based refraction correction. Huang et al. [5] utilized Mask R-CNN to precisely segment fish body contours from stereo images, integrating this with 3D reconstruction technology to achieve accurate length estimation and demonstrating the capabilities of deep learning in complex aquaculture scenarios.

Contemporary research prioritizes the comprehensive optimization of algorithmic accuracy, efficiency, and scene adaptability. The YOLO (You Only Look Once) series algorithms, known for their excellent balance between real-time performance and detection accuracy, have emerged as a cornerstone for non-contact, high-throughput object detection and are extensively employed in visual perception tasks across agriculture, industry, and ecological monitoring [6]. In fish measurement, Fernandes et al. [7] used a SegNet-based model to distinguish the body and fins of tilapia, though its versatility was limited by off-water measurement requirements. To address species-specific challenges, Feng et al. [8] proposed the YOLMA system, introducing an Asymptotic Feature Pyramid Network (AFPN) to address the challenge of measuring the transparent pectoral fins of large yellow croaker (*Larimichthys crocea*) and achieving automatic quantification of the pectoral fin length to pelvic fin length (PFL/PEL) ratio for the first time. Cong et al. [9] combined DeepLabV3+ and CatBoost regression models to estimate the body length and weight of pearl gentian grouper with high accuracy (R^2^ = 0.987). Despite these advances, existing studies predominantly focus on static, single-target, or ex situ scenarios. In dynamic, high-density aquaculture environments—characterized by frequent occlusions, crossing events, and variable water quality—continuous, stable, and high-precision body-length monitoring of species such as *M. salmoides* remains challenging due to difficulties in small-target detection and frequent identity switches in dense scenes. Consequently, there is a pressing need to develop advanced algorithms that concurrently address real-time performance, robustness, and tracking stability.

In view of the above limitations, this paper proposes an improved YOLOv8-based multi-target tracking framework for dynamic body length monitoring of *M. salmoides* in intensive recirculating aquaculture systems (RAS). The proposed system addresses three specific challenges: (1) high fish density leading to frequent occlusions and identity switches; (2) variable illumination and water turbidity degrading image quality; and (3) the need for precise conversion from pixel measurements to physical body length, accounting for water–air refraction and fish pose variation. The main contributions of this study are as follows:

(1) A shallow feature fusion mechanism (FPN-Aug) is proposed to enhance the network’s perception of small-sized fish targets. By fusing shallow high-resolution features from the backbone network, this mechanism preserves edge and contour detail information and significantly improves detection accuracy for individuals at long distances or of small body size. This component represents a novel adaptation of feature pyramid architecture specifically designed for high-density aquaculture monitoring.

(2) A lightweight MobileNetV3_ReID module is adapted and integrated into the tracking pipeline to extract discriminative appearance features for each detected fish. The module achieves efficient identity discrimination with low parameter count, providing a reliable appearance basis for multi-target continuous tracking. While MobileNetV3 is an established architecture, its adaptation for fish ReID and integration with the tracking framework represents a targeted contribution for aquaculture applications.

(3) A hybrid matching strategy combining IoU (Intersection over Union), appearance feature cosine similarity, and motion consistency is constructed. When associating detection boxes with trajectories, spatial position and visual similarity are jointly considered, which effectively suppresses identity switching and ensures the continuity and reliability of individual body-length time series. The dynamic threshold adjustment and exponential moving-average feature update are novel extensions tailored to the high-density, high-interaction conditions of intensive aquaculture.

This study is expected to provide a feasible technical scheme for non-contact and continuous monitoring of fish growth status in intensive recirculating aquaculture environments.

## 2. Materials and Methods

### 2.1. Dataset

#### 2.1.1. Experimental System and Data Acquisition

The experiment was carried out in the recirculating aquaculture system (RAS) of the fish-vegetable symbiotic AI factory at the Chongqing Academy of Agricultural Sciences. The experimental subjects were freshwater-cultured *Micropterus salmoides*. To ensure environmental stability during data collection, a single circular culture pond (diameter approximately 5 m, water depth 1.6 m) was selected for video capture. Water temperature was maintained at 22–26 °C, dissolved oxygen concentration above 6 mg/L, and pH at 7.0 ± 0.3, consistent with industrial aquaculture standards for this species.

**Figure 1:**
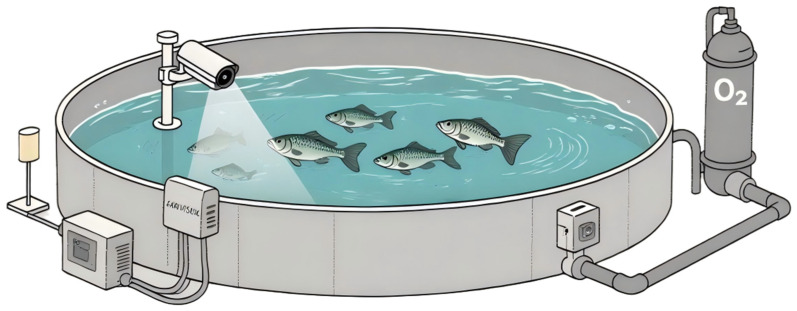
Experimental device and equipment.

The video data acquisition system comprised an overhead high-definition network camera (Hikvision DS-2CD2347G1-L), LED fill light, and a desktop workstation (Intel Core i7-14650HX, 32 GB DDR5 RAM, NVIDIA GeForce RTX 4060 8 GB GPU, Windows 11 Home Chinese Edition). Importantly, the camera was mounted approximately 1 m above the water surface and oriented at a 60° tilt angle relative to the water surface, capturing top-view video of fish swimming in the water column. This configuration is distinct from true underwater imaging, as the camera resides in the air and views fish through the water–air interface. The video stream was transmitted to the workstation via local area network and recorded at 1920 × 1080 resolution and 30 fps using OpenCV 4.11.0 with Python 3.8. All detection and tracking experiments were conducted on this workstation using PyTorch 2.4.1 with CUDA 11.8, with inference batch size fixed at 1 to simulate real-time single-frame processing. The experimental equipment is shown in Figure 1.

To construct a dataset covering diverse visual conditions, video was collected from 100 clips recorded over a 24 h period, capturing samples with varying swimming postures, fish densities, and illumination conditions. Video processing tools were used to extract 1 frame per 30 frames (1 fps sampling) from the original video for image sampling. All images were manually screened to exclude samples with severe motion blur, overexposure, or complete occlusion, yielding a total of 1800 valid images. The dataset was divided into training (1260 images), validation (360 images), and test (180 images) sets according to a 7:2:1 ratio [10].

It should be noted that the train/validation/test split was performed by random sampling across all extracted frames. While this ensures statistical consistency in scene distribution, adjacent frames in the original video sequence may fall into different subsets, meaning training and test images may share similar illumination conditions and individual fish appearances. This represents a limitation of the current dataset, which was collected from a single pond over a single 24 h period.

#### 2.1.2. Data Augmentation

To improve the robustness and generalization capability of the model under the imaging conditions encountered in intensive recirculating aquaculture systems, this study designed a multi-dimensional data augmentation strategy integrating geometric transformations, color adjustments, and domain adaptation mechanisms. The strategy addresses challenges including uneven illumination, refractive distortion at the water–air interface, background interference from pond infrastructure, and variable target pose during the aquaculture imaging process [11].

Table 1 summarizes the augmentation conditions and their sources. Conditions present in the original collected data included variable fish density (15–35 individuals per frame), natural illumination variation across the 24 h collection period, and moderate turbidity (20–40 NTU). Synthetically generated conditions included simulated extreme turbidity (>50 NTU), severe occlusion events, and cross-illumination scenarios not fully captured during the collection window.

At the geometric level, random rotation (±30°) was introduced to simulate the attitude diversity of fish during free swimming, and asymmetric scaling (0.8× to 1.2×) was applied to simulate scale changes at different observation distances. Elastic deformation was used to model the local distortion caused by water refraction at the water–air interface, improving the model’s tolerance to geometric interference. Random cropping operations simulated occlusion and boundary truncation between individuals, enhancing robustness to partial information loss. A multi-scale Mosaic augmentation strategy was also employed, in which four training images were randomly spliced into a single input sample, enriching context information and enhancing spatial resolution for dense targets.

At the color and illumination level, in addition to standard ImageNet normalization, channel-specific gain adjustments were applied to account for the typical green body coloration of *M. salmoides*: the blue channel was moderately enhanced (+15%) to improve contrast between fish body and background, while the red channel was suppressed (−10%) to reduce spectral interference from sediment and artificial structures at the pond bottom. To narrow the domain gap between annotated training data and real aquaculture environments, a lightweight anti-disturbance generation module was introduced to simulate sensor noise and dynamic environmental interference [12]. Combined with CycleGAN, style-transfer augmentation across devices and illumination conditions was realized, improving model transferability under different imaging configurations.

For task-oriented enhancement, a Gaussian heatmap (σ = 3 pixels) of the rostral and caudal positions was generated for each fish during labeling, providing a supervisory signal for subsequent keypoint regression. A body-length labeling error compensation mechanism was also constructed, in which Euclidean distance is dynamically corrected according to fish body tilt angle, alleviating systematic measurement error caused by pose variation.

The quantitative validation of the augmentation strategy is presented in Section 3.3.1 to maintain a clear separation between method description and results reporting.

#### 2.1.3. Image Annotation

To improve labeling efficiency and ensure annotation quality, an automated image labeling workflow was constructed using the T-Rex Label platform for efficient and standardized data processing.

In this workflow, the platform’s pre-trained model was used to automatically detect all 1800 images and generate initial bounding boxes, uniformly labeled with the category ‘Largemouth Bass’. The quantitative accuracy of the pre-labeling stage (e.g., IoU distribution relative to manual annotations) and the efficiency gains of the collaborative workflow are reported in Section 3.3.1 alongside other preliminary validations. In challenging scenes such as high-density fish schools, low visibility, or strong light reflection, significant positioning deviations, missed detections, and false detections remained—caused by interference from fish shadows and overlapping individuals—necessitating manual intervention to improve annotation accuracy.

To ensure data fidelity, a labeling team of three professionals with aquatic biology backgrounds was assembled to review and correct the pre-labeling results frame by frame. Corrections focused on fine adjustment of detection boxes to match real fish contours, identification of missing individuals in dense areas, and elimination of misidentified non-target distractors. The revised labels were independently reviewed by a second expert to ensure consistency of annotation criteria and reliability of results, yielding a high-quality training dataset.

**Figure 2:**
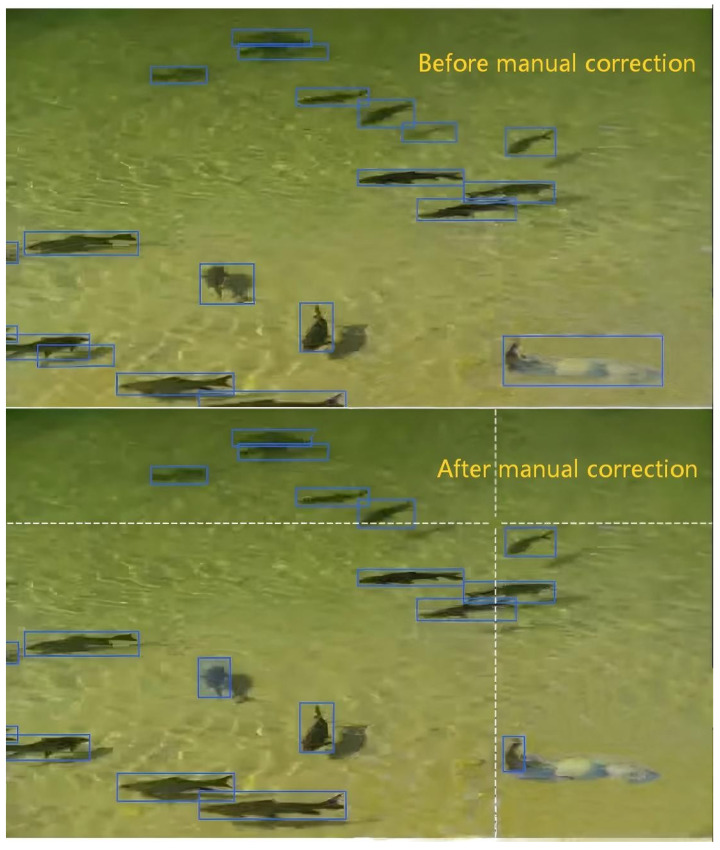
Comparison of pre-labeling and manual correction effects.

A comparison of pre-annotated and manually corrected bounding boxes is shown in Figure 2, illustrating the method’s effectiveness in addressing complex visual challenges such as edge blur and partial occlusion.

### 2.2. Geometric Vision-Based Body Length Measurement Model

The precision of body length estimation is significantly affected by light refraction at the water–air interface and the stochastic nature of fish postures. Establishing a precise correspondence between 2D image space and 3D physical space requires a geometric measurement framework that integrates camera calibration, optical refraction compensation, and pose-adaptive projection. This framework enables real-time transformation of observed pixel lengths to true biological dimensions.

The measurement pipeline operates as follows: (1) camera intrinsic and extrinsic parameters are obtained through calibration (Section 2.2.1); (2) for each detected fish keypoint (head and tail), the observed pixel coordinates are corrected for water–air refraction using Snell’s law (Section 2.2.2); (3) the corrected 2D coordinates are back-projected to 3D world coordinates using an estimated fish swimming depth (Section 2.2.3); and (4) the 3D Euclidean distance between head and tail keypoints is computed and corrected for fish pose (tilt angle) to obtain the final body length estimate (Section 2.2.4). Steps (2) and (3) are applied jointly at inference time: the refraction-corrected observation direction from Section 2.2.2 is intersected with the estimated fish depth plane (Section 2.2.3) to obtain accurate 3D keypoint positions.

#### 2.2.1. Camera Calibration and Coordinate Systems

The overhead camera was calibrated using Zhang’s method [13] to obtain the intrinsic matrix K and distortion coefficients D, as defined in Formula (1):

(1) $$\begin{array}{c} K=\left[\begin{array}{ccc} f_{x} & 0 & c_{x}\\ 0 & f_{y} & c_{y}\\ 0 & 0 & 1 \end{array}\right],\;D=[k_{1},k_{2},p_{1},p_{2},k_{3}] \end{array}$$

where $f_{x}$ and $f_{y}$ denote the focal lengths in pixels, and ($c_{x}$,$c_{y}$) represent the coordinates of the principal point. Three coordinate systems were established to describe the geometric relationships: the world coordinate system $O_{w}-X_{w}Y_{w}Z_{w}$, with the $Z_{w}$-axis pointing vertically upward, and the water surface located at $Z_{w}$ = 0; the camera coordinate system $O_{c}-X_{c}Y_{c}Z_{c}$, where the $Z_{c}$-axis aligns with the optical axis, and the image pixel coordinate system $O_{i}-uv$. The transformation from the world coordinate system to the camera coordinate system is governed by the extrinsic matrix [R|t], where t is the translation vector and R is the rotation matrix describing the camera pose. The rotation matrix is formulated as Formula (2):

(2) $$\begin{array}{c} R=R_{y}(\theta )\cdot R_{x}(\phi )\cdot R_{z}(\psi ) \end{array}$$

where $\theta =60^{\circ }$ represents the pitch angle, while ϕ and ψ denote the roll and yaw angles, respectively, which were determined through calibration board experiments. The translation vector $t=[t_{x},t_{y},t_{z}{]}^{T}$, T defines the position of the camera optical center within the world coordinate system.

#### 2.2.2. Water–Air Interface Refraction Correction

Light rays undergo refraction at the water–air interface of the aquaculture pool. If modeled using a standard pinhole camera model, this phenomenon causes objects to appear closer and larger than their actual physical dimensions. To mitigate this distortion, we employ a correction method based on Snell’s Law.

When the camera captures images at an oblique angle of 60°, the incident angle of light traveling from water to air varies with pixel position, rendering the refraction effect non-radially symmetric. Consequently, we adopt a 3D vector-based refraction model for correction.

Let $v_{c}$ be the unit observation vector in the camera coordinate system corresponding to a pixel point p(u,v), defined as Formula (3):

(3) $$v_{c}=\frac{K^{-1}\cdot [u,v,1{]}^{T}}{\left\|K^{-1}\cdot [u,v,1{]}^{T}\right\|}$$

This vector is rotated by the extrinsic parameters to obtain the observation direction $v_{w}$ in the world coordinate system:$\begin{array}{c} v_{w}=R^{T}\cdot v_{c} \end{array}$.

According to Snell’s Law, the refraction at the water–air interface satisfies Formula (4):

(4) $$\begin{array}{c} n_{a}\sin(\theta_{a})=n_{w}\sin(\theta_{w}) \end{array}$$

where $n_{a}\approx 1.00$ and $n_{w}\approx 1.33$ are the refractive indices of air and water, respectively, and $\theta_{w}$ and $\theta_{a}$ denote the incident angle in water and the refraction angle in air, respectively.

Let the normal vector of the water surface be $n=[0,0,1{]}^{T}$ (pointing vertically upward). The incident angle $\theta_{w}$ is calculated via the vector dot product, as shown in Formula (5):

(5) $$\begin{array}{c} \cos(\theta_{w})=|v_{w}\cdot n|,\theta_{w}=\arccos(|v_{w}\cdot n|) \end{array}$$

The refracted direction vector ${v^{\prime }}_{w}$ is then computed as Formula (6):

(6) $$\begin{array}{c} {v^{\prime }}_{w}=\frac{n_{w}}{n_{a}}v_{w}+\left(\frac{n_{w}}{n_{a}}\cos(\theta_{w})-\sqrt{1-{\left(\frac{n_{w}}{n_{a}}\right)}^{2}(1-\cos^{2}(\theta_{w}))}\right)n \end{array}$$

This step corrects the observation direction to align with the true underwater light propagation path.

**Figure 3:**
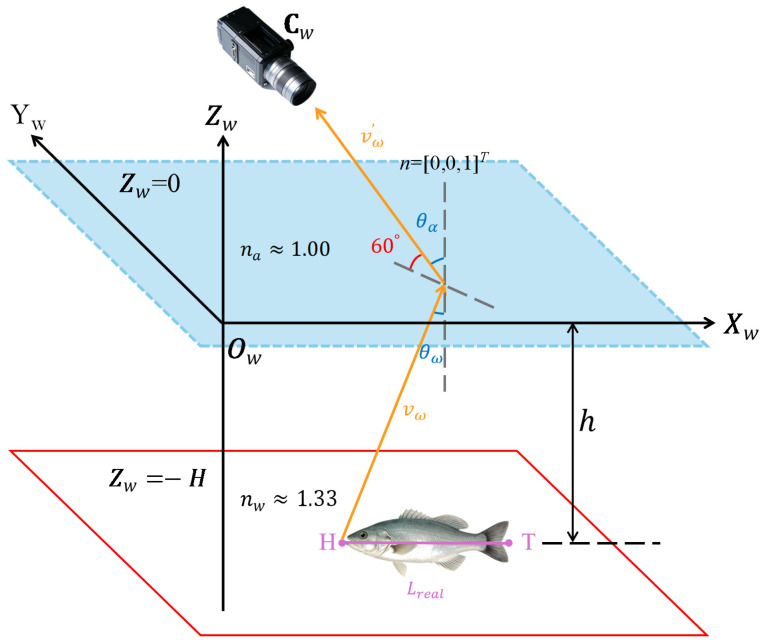
Schematic diagram of water–air interface refraction correction.

For a keypoint detected at pixel coordinates (u,v), we first calculate its normalized radial distance $r=\sqrt{(u-c_{x}{)}^{2}+(v-c_{y}{)}^{2}}$. The apparent angle $\theta_{a}$ is derived as $\theta_{a}=\arctan(r/f_{eff})$, where $f_{eff}$ is the effective focal length. The corrected radial distance $r_{corr}$ in the water medium is calculated according to Formula (7):

(7) $$\begin{array}{c} r_{corr}=h\cdot \tan\left(\arcsin\left(\frac{n_{a}}{n_{w}}\sin\left(\arctan\frac{r}{f_{eff}}\right)\right)\right) \end{array}$$

where h represents the vertical depth of the fish below the water surface. At inference time, h is estimated from the pond geometry: given a total water depth of 1.6 m and the observation that *Micropterus salmoides* typically occupies the middle water column during cruising behavior, a nominal depth of h≈0.8 m is assumed. This assumption is supported by our empirical observations from 48 h of overhead video recordings, in which the majority (>75%) of fish detections occurred within the central 0.6–1.0 m depth band. This approximation introduces a depth estimation error that affects the accuracy of the refraction correction. A sensitivity analysis of body-length error with respect to depth estimation error is provided in Section 2.2.3. A schematic diagram of this projection process is illustrated in Figure 3.

#### 2.2.3. 3D Back-Projection and Depth Estimation

Due to oblique shooting, the actual water depth corresponding to different positions in the image varies. Let the pool bottom equation in the world coordinate system be $Z_{w}=-H$ (H is the pool depth), and the fish swimming depth be $Z_{w}=h$ (h<H).

For the corrected observation direction ${v^{\prime }}_{w}$ and the camera optical center position $C_{w}$ (computed from t), the intersection point $P_{w}$ of the light ray with the water depth plane is shown in Formula (8):

(8) $$P_{w}=C_{w}+\lambda \cdot {v^{\prime }}_{w},\lambda =\frac{-h-C_{w,z}}{{v^{\prime }}_{w,z}}$$

where $C_{w,z}$ and ${v^{\prime }}_{w,z}$ are the Z components of the camera position and observation vector, respectively.

This yields the 3D world coordinates of the fish keypoints $P_{head}^{world}$ and $P_{tail}^{world}$.

A sensitivity analysis of body-length error with respect to depth-estimation error is provided in Section 3.3.1.

#### 2.2.4. Pose-Aware Body Length Calculation and Temporal Smoothing

When the fish body is tilted relative to the camera optical axis, the Euclidean distance between the head (H) and tail (T) coordinates output by the detection network in the image plane underestimates the true body length. Let $p_{h}$ and $p_{t}$ denote the 2D coordinates of the head and tail, respectively, after refraction correction. 

Assuming that the fish primarily swims within a horizontal plane at depth $Z_{\mathrm{depth}}$ (estimated based on the pond geometry), we back-project these points into the 3D world space as follows in Formula (9):

(9) $$\begin{array}{c} P_{world}=Z_{depth}\cdot K^{-1}\cdot \tilde{p} \end{array}$$

where $\tilde{p}=[u,v,1{]}^{T}$ represents the homogeneous coordinate vector, and K is the camera intrinsic matrix.

The instantaneous body length $L_{t}$ is then computed in Formula (10) as the Euclidean distance in 3D space:

(10) $$L_{t}=\|P_{head}^{world}-P_{tail}^{world}{\|}_{2}$$

To account for potential minor vertical undulations (characterized by a pitch angle α), we introduce a pose correction factor γ=1/cos(α). The angle α is estimated dynamically from the aspect ratio of the detection bounding box or the slope of the trajectory across consecutive frames. The final corrected body length $L_{corr}$ is calculated as $L_{corr}=L_{t}\cdot \gamma$.

Furthermore, to suppress jitter induced by detection noise and water turbulence, we apply a weighted moving average (WMA) over a sliding window of N=10 frames, as shown in Formula (11):

(11) $$\begin{array}{c} L_{final}=\frac{\sum_{i=t-N+1}^{t}w_{i}\cdot L_{corr}^{\left(i\right)}}{\sum_{i=t-N+1}^{t}w_{i}},\;w_{i}=i-(t-N) \end{array}$$

where recent frames are assigned higher weights ($w_{i}$). This temporal smoothing strategy ensures that each tracked identity yields a stable and biologically plausible body length estimate.

To validate the accuracy of body length estimation, a dedicated experiment was conducted as follows: (1) fifteen *Micropterus salmoides* individuals were randomly captured from the culture pond. (2) Each individual was measured using a digital caliper (precision: ±0.1 mm) along the standard total-length axis (tip of snout to end of caudal fin); three repeated measurements were taken per individual, and the median value was recorded as the ground-truth length. (3) The 15 individuals were then fitted with differently colored ribbons at the anterior base of the caudal fin and released back into the pond. (4) During the subsequent 48 h video recording period, the automated system automatically identified and tracked these individuals, and the body length of each tracked individual was computed using the aforementioned procedure. (5) For each manually measured fish, the corresponding automated measurement result was determined by matching the manual measurement timestamp with the temporal overlap window of the tracking trajectory and the caudal ribbon color. (6) The predicted body length for each individual was computed as the weighted moving average (WMA) smoothed estimate (Formula (11), window W = 30 frames, decay factor λ = 0.9) over a stable tracking period.

The mean absolute error (MAE) was 1.35 cm, the root mean square error (RMSE) was 1.58 cm, and the body length error (BLE) was 5.2 ± 1.8% across the 15 validation fish (body length range: 18.5–32.7 cm, mean: 25.8 cm). The Pearson correlation coefficient between predicted and manually measured lengths was r = 0.972 (*p* < 0.001). These results indicate that the measurement pipeline achieves accuracy adequate for aquaculture growth monitoring applications, where typical management decisions tolerate length estimation errors of 5–10%.

### 2.3. Construction of an Intelligent Body-Length Monitoring Model for Micropterus salmoides

#### 2.3.1. Overall Framework Design

In order to realize non-contact and high-precision dynamic monitoring of the length of *Micropterus salmoides*, we propose a multi-target tracking system that integrates improved target detection, lightweight re-identification, and multi-modal data association. The system adopts a typical tracking-by-detection (TBD) paradigm and consists of four core modules: an improved YOLOv8 target detector, a MobileNetV3_ReID feature extractor, a multi-modal data association module, and a body length measurement and error compensation mechanism [14].

**Figure 4:**
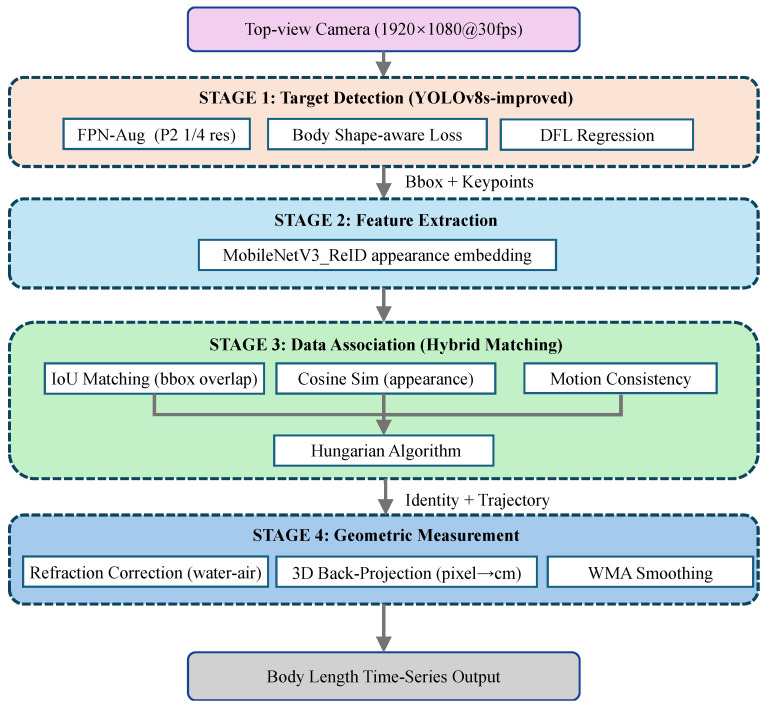
End-to-end system workflow for non-contact body length monitoring of *Micropterus salmoides*.

The system obtains the bounding box of the fish body in each frame image by improving the YOLOv8 model and uses the lightweight ReID network to extract the appearance features of the target. On this basis, a cost matrix integrating motion and appearance information is constructed. The improved Hungarian algorithm is used to achieve cross-frame identity matching. Combined with calibration parameters and attitude correction strategies, the continuous estimation of individual fish body length is realized. The framework, designed to balance detection accuracy, tracking stability, and computational efficiency, is shown in Figure 4, making it suitable for long-term growth monitoring tasks in aquaculture environments.

#### 2.3.2. YOLOv8 Infrastructure

**Figure 5:**
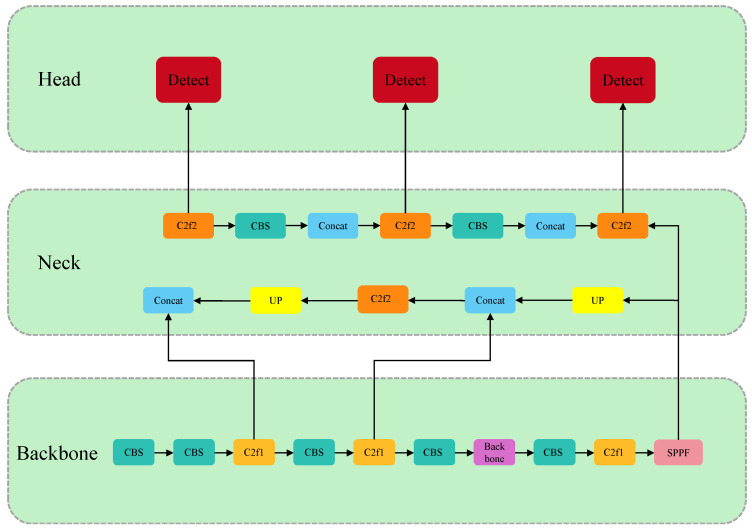
YOLOv8 network architecture.

YOLOv8 is a single-stage object detection model released by Ultralytics in 2023. YOLOv8 inherits the efficient structure of YOLOv5 and optimizes the network design, training strategy, and task scalability. Its network architecture is shown in Figure 5, which is mainly composed of three parts: Head, Neck, and Backbone [15].

The backbone network adopts CSPDarknet structure, combined with C2 f module and SPPF (Spatial Pyramid Pooling Fast) module, to effectively enhance gradient flow and improve multi-scale feature expression [16]. The Neck part is based on the PAN-FPN (Path Aggregation Network with Feature Pyramid Network) structure, which enhances the feature fusion of small targets by bottom-up and top-down bidirectional path aggregation. The detection head abandons the traditional Anchor-based mechanism and adopts the Anchor-free paradigm to directly predict the coordinates of the target center point and the bounding box, which simplifies the model structure and improves the generalization performance [15].

In terms of training strategy, YOLOv8 introduces the task-aligned assigner positive and negative sample allocation mechanism, dynamically aligns classification and positioning tasks, and alleviates the task mismatch problem in static matching. The loss function uses a combination of CIoU loss and distribution focal loss (DFL) to optimize the bounding box regression accuracy [17]. In addition, the model supports advanced data augmentation techniques such as Mosaic and MixUp and adopts the cosine annealing learning rate scheduling strategy to significantly improve the convergence speed and robustness [18].

In view of its high precision and real-time performance in complex scenes, YOLOv8 has been widely used in aquatic intelligent monitoring tasks such as underwater target detection and dense fish swarm recognition, which provides a reliable detection basis for experiments.

#### 2.3.3. Model Improvement for Individual Detection of *Micropterus salmoides*

**Figure 6:**
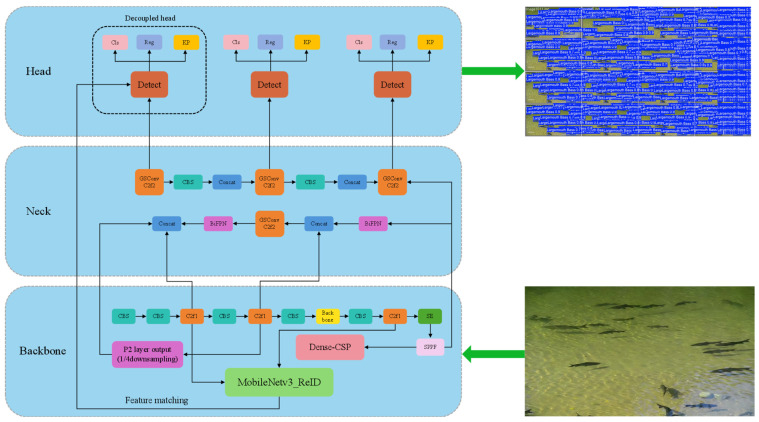
Special detection model for body length monitoring of *Micropterus salmoides*.

Aiming at the problems of a high proportion of small targets, uneven illumination, and turbid water quality in fish detection, we propose three key improvements based on YOLOv8s and then construct a special detection model for *Micropterus salmoides* body length monitoring, as shown in Figure 6.

In order to improve the model’s ability to perceive long-distance or small-size individuals, especially to accurately identify small structures such as fish heads in high-density scenes, a shallow feature injection path at the P2 level (resolution is 1/4 of the input) is introduced and integrated into the FPN feature fusion structure [14]. This design enhances the transmission efficiency of low-level high-resolution features in the detection process, effectively alleviates the attenuation of detailed information in the deep network, and significantly improves the positioning accuracy of small targets.

On the premise of ensuring the detection performance, the efficiency of the model is further optimized, and the detection head is lightweight reconstructed. Grouped spatial convolution (GSConv) is used to replace the traditional convolution operation, which reduces the parameter redundancy while maintaining the feature expression ability, reduces the parameter quantity of the detection head by 18%, and improves the inference efficiency and resource adaptability of the model [19].

To further enhance the biological plausibility and spatial localization accuracy of bounding boxes, we introduce a Body Shape-aware Loss function. This mechanism incorporates an auxiliary keypoint detection branch to supervise the model in learning the spatial distribution of key anatomical landmarks, specifically the head, tail, and dorsal fin. By integrating such prior knowledge of fish morphology, this constraint guides the detection boxes to align more closely with the true fish body contours. Consequently, it significantly improves localization stability and geometric consistency, particularly under challenging conditions such as partial occlusion or severe pose variations.

The keypoint detection branch employs a 3-layer lightweight convolutional structure. Each layer consists of a 3 × 3 convolution, followed by batch normalization (BN) and the SiLU activation function. The channel dimensions progressively decrease from 256 to 128 and finally to 64. The branch culminates in a 1 × 1 convolution layer that outputs keypoint heatmaps. For each detected bounding box, the network predicts the normalized offsets $\left(\delta_{x},\delta_{y}\right)$ for the three keypoints relative to the box center. This auxiliary branch introduces approximately 0.3 MB of additional parameters, keeping the total model parameter count within 1.91 MB.

The total loss function of the model is defined as a weighted combination of multiple terms:

$$\begin{array}{c} L_{\mathrm{total}}=\lambda_{\mathrm{box}}L_{\mathrm{box}}+\lambda_{\mathrm{obj}}L_{\mathrm{obj}}+\lambda_{\mathrm{cls}}L_{\mathrm{cls}}+\lambda_{\mathrm{size}}L_{\mathrm{size}}+\lambda_{\mathrm{kp}}L_{\mathrm{kp}} \end{array}$$

where $L_{\mathrm{box}}$ denotes the CIoU loss for bounding box regression error; $L_{\mathrm{obj}}$ represents the binary cross-entropy (BCE) loss for object confidence prediction; $L_{\mathrm{cls}}$ is the category classification loss. Since this study focuses on single-class detection, its weight is set to zero ($\lambda_{\mathrm{cls}}$ = 0); $L_{\mathrm{size}}$ is the body length regression loss, employing Smooth L1 loss to supervise the deviation between predicted and ground-truth body lengths; $L_{\mathrm{kp}}$ is the keypoint detection loss, calculated using weighted mean squared error (MSE) to measure the discrepancy between predicted keypoint positions and annotated heatmaps.

The weighting coefficients are optimized via grid search on the validation set and determined as $\lambda_{\mathrm{box}}$ = 0.05, $\lambda_{\mathrm{obj}}$ = 0.58, $\lambda_{\mathrm{cls}}$ = 0, $\lambda_{\mathrm{size}}$ = 0.10, and $\lambda_{\mathrm{kp}}$ = 0.37.

The model training uses an optimal configuration with an initial learning rate of 0.01, combined with a cosine annealing scheduler to achieve dynamic attenuation of the learning rate [14]. Compared with the fixed learning rate strategy, this method improves the convergence speed of the model by about 25% and enhances the stability of the training process. At the same time, the early stopping mechanism (patience = 30) is set up, and mAP@0.5:0.95 on the verification set is used as the monitoring index to avoid overfitting and ensure the generalization ability of the model.

In order to enhance the robustness of the model in the real aquaculture environment, the data enhancement strategy further integrates specific perturbations on the basis of the standard Mosaic and MixUp: simulating the refraction effect of water with a probability of 40%, introducing algae suspended matter noise (density ≤ 15%) to enhance the adaptability to turbid water quality, and applying 1.1 to 1.3 times the dynamic gain of the blue channel to simulate the physical characteristics of spectral attenuation under different water depth conditions.

**Figure 7:**
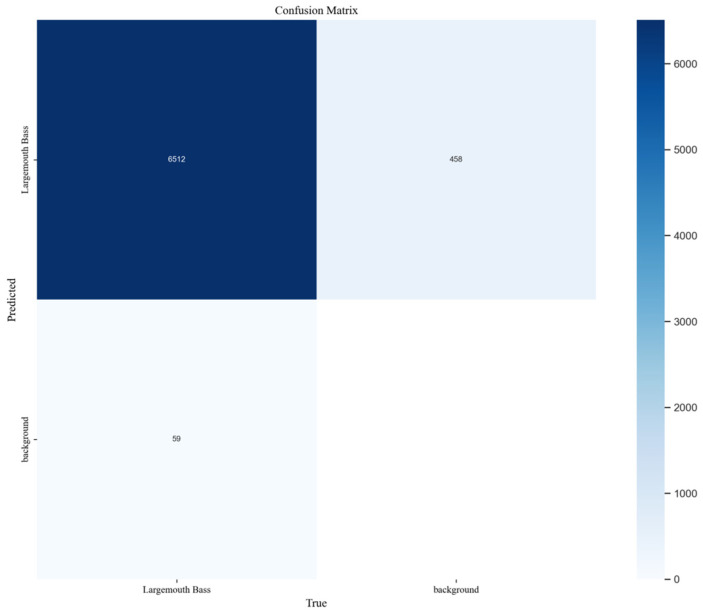
Visualization of a confusion matrix.

The test results show that the above improvements significantly improve the comprehensive performance of the model on the self-built *Micropterus salmoides* dataset. The visualized confusion matrix is shown in Figure 7. The accuracy of category recognition is significantly improved, and the body length measurement error (BLE) is effectively controlled. At the same time, in the high-density fish scene, the false detection rate and ID switching frequency are significantly reduced, which verifies the effectiveness and practicability of the proposed method in complex breeding visual tasks.

#### 2.3.4. MobileNetV3_ReID Lightweight Re-Identification Module

**Figure 8:**
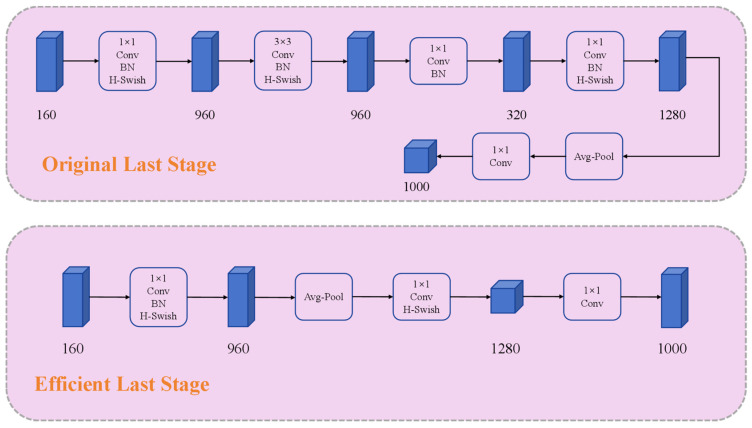
Network architecture of MobileNetV3_ReID model.

In order to improve the identity consistency in multi-target tracking, we construct a lightweight re-identification model based on MobileNetV3-large, named MobileNetV3_ReID. The model architecture is shown in Figure 8, which is dedicated to extracting the appearance features of fish [20].

The model takes the pre-trained weights on ImageNet as the initialization parameter and fine-tunes on this basis to adapt to the visual characteristics of fish. After the input image was standardized (mean: [0.485, 0.456, 0.406], standard deviation: [0.229, 0.224, 0.225]), the resolution was adjusted to 224 × 224 as the network input.

The original classification layer is replaced by a three-layer fully connected structure containing batch normalization and the ReLU activation function. The hidden layer dimensions are 512 and 256 in turn, and finally mapped back to the 512-dimensional feature space [21]. This structure reduces the overall parameter of the model by 46% while compressing the feature representation dimension, which significantly improves the computational efficiency. The output features are normalized and mapped to the unit hypersphere space, which facilitates the subsequent appearance similarity measurement across frames or perspectives through cosine similarity.

In terms of deployment optimization, in order to improve the inference efficiency of the model, a number of lightweight inference strategies are adopted: in the inference stage, the statistical parameters of the batch normalization layer are fixed, the gradient calculation is disabled to reduce the computational graph overhead, and the fusion operation of the convolution layer and the batch normalization layer is implemented to further accelerate the forward propagation process. To ensure consistent experimental conditions, all models were evaluated using PyTorch’s automatic mixed-precision (AMP) framework (torch.cuda.amp.autocast()), which automatically converts applicable operations to FP16 precision on compatible NVIDIA GPUs. It should be noted that the original experiments were already conducted with AMP enabled. This study employs framework-level AMP, not a platform-specific optimized deployment engine such as TensorRT. Therefore, the reported throughput data should be interpreted as a preliminary comparative assessment under the described desktop-GPU configuration, rather than as evidence of optimized reduced-precision deployment readiness. Aiming at the problem that the extremely small target may introduce noise features, a robustness enhancement mechanism is designed: when the target size corresponding to the detection frame is less than 10 pixels, the model returns a zero vector to avoid the interference of invalid appearance features on data association; at the same time, the geometric transformation operation in the image preprocessing process is captured to ensure the stability of the system in the case of abnormal input or low quality detection box.

The evaluation results on the FishReID benchmark dataset are shown in Table 2. The model achieves a single-frame inference delay of 9.8 ms on the RTX4060 GPU, achieving 74.6% mAP and 87.3% Rank-1 accuracy, which are 6.4 and 5.2 percentage points higher than the original MobileNetV3 architecture, respectively. Especially under complex water conditions with turbidity higher than 50 NTU, the cross-camera re-identification performance is significantly improved, and the accuracy of Rank-1 is increased by 8.7%, which is better than the backbone network with larger parameters, such as ResNet50. The comprehensive performance verifies the good balance between accuracy and efficiency of MobileNetV3_ReID [22].

#### 2.3.5. Multi-Modal Data Association and Tracker Design

The completed multi-target tracking system adopts the integrated architecture of detection and tracking, aiming to improve the accuracy and real-time performance of the *Micropterus salmoides* body length monitoring task. The system consists of three core modules: a target detection module based on improved YOLOv8, a lightweight MobileNetV3_ReID feature-extraction module, and a data-association module integrating multi-modal information. During the operation, the target detection module outputs the bounding box position of the fish body, and the feature extraction module synchronously generates a highly discriminative appearance embedding vector for each detection instance. The multi-modal data association mechanism comprehensively utilizes space, appearance, and motion information to achieve cross-frame identity matching and stable trajectory maintenance. In the data association link, the improved Hungarian algorithm is used as the matching core, and its cost matrix is composed of spatial overlap and appearance similarity [23], as shown in Formula (12):

(12) $$\mathrm{Cij}=\alpha \cdot \mathrm{IoU}(b_{i}^{t},b_{j}^{t-1})+(1-\alpha )\cdot \cos(\mathrm{fi},f_{j})$$

Among them, α= 0.5 is the balance coefficient determined on the verification set by grid search, which is used to coordinate the contribution of spatial overlap and appearance similarity in the matching process to achieve the optimal fusion of the two. In order to improve the adaptability of the algorithm in high-density fish swarm scenarios, the system introduces multiple mechanisms to collaboratively optimize data association performance.

The dynamic matching threshold strategy adaptively adjusts the matching threshold according to the historical IoU changes in the sliding window [24], as shown in Formula (13):

(13) $$\tau_{t}=\tau_{0}+\eta \cdot \frac{1}{w}{\sum k=t-w}^{t-1}I(\mathrm{IoUk}>\tau_{\mathrm{iou}})$$

Here, w = 10 is the size of the observation window and η is the learning rate. The strategy can dynamically adjust the matching sensitivity according to the complexity of the environment, effectively suppress the error correlation when the fish swarm interacts intensively, and effectively improve the ID retention rate.

The update of the appearance feature of the target adopts the exponential moving average (EMA) strategy to take into account the stability of the feature representation and the adaptability to the attitude change. The update method is shown in Formula (14):

(14) $$\mathrm{ft}=\beta \mathrm{ft}-1+(1-\beta )f_{\mathrm{new}}$$

By adjusting the β value, a good balance between historical features and new observations can be achieved, which not only retains the ability of identity discrimination, but also avoids the mismatch caused by feature pollution.

In the aspect of trajectory management, an adaptive vanishing detection window mechanism based on frame rate is proposed, as shown in Formula (15):

(15) T=⌈0.5×fps⌉

The mechanism dynamically sets the trajectory retention time according to the actual frame rate of the video. Compared with the fixed-time strategy, it is more in line with the time-resolution characteristics of different acquisition devices, effectively reduces the risk of trajectory error continuation, and can significantly reduce the error-tracking rate.

The above mechanisms work synergistically to improve the identity consistency and tracking robustness of the system in complex aquaculture environments.

In order to improve the overall operating efficiency of the system, three optimization measures are implemented. The validity verification mechanism of the detection frame filters invalid inputs and reduces redundant calculations by prejudging the rationality of the target size and position, as shown in Formula (16):

(16) $$V(b)=I(w\geq 10)\cdot I(h\geq 10)\cdot I(\frac{\max(w,h)}{\min(w,h)}<3.5)$$

This mechanism effectively reduces the amount of invalid calculations. At the same time, at the reasoning level, the feature extraction speed of MobileNetV3_ReID is improved by the fusion technology of the convolution layer and the batch normalization layer [25].

Facing the special challenges of the aquaculture environment, the system integrates multiple robust designs. The image enhancement preprocessing module can effectively suppress the visual degradation caused by turbid water (>50 NTU) and ensure a high level of tracking accuracy under this condition. At the same time, the motion consistency constraint is introduced, as shown in Formula (17):

(17) $$\psi \mathrm{ij}=\frac{\exp(-|\mathrm{vi}-\mathrm{vj}|/\sigma )}{\mathrm{IoUij}}$$

It is used to filter false associations that do not conform to the laws of biological motion and improve tracking accuracy in dense scenes. For targets lost short-term due to occlusion or blurring, the system employs a prediction mechanism based on the kinematic model for trajectory compensation, as shown in Formula (18):

(18) $$\hat{x}t=A\mathrm{xt}-1+N(0,Q)$$

Among them, A contains the fish motion characteristic parameters, such as acceleration and steering preference, which are included to ensure the spatio-temporal continuity of the trajectory.

**Figure 9:**
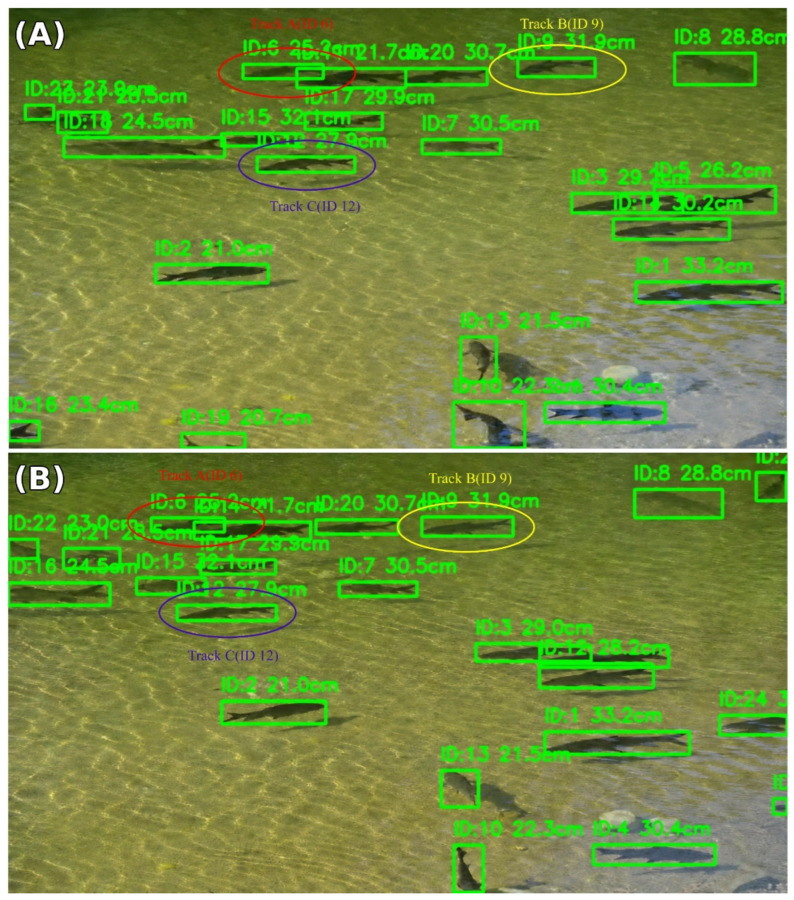
Qualitative tracking results in two temporally close video frames under dense aquaculture conditions. (**A**) Tracking result at frame t. (**B**) Tracking result at frame t + 30. The colored ellipses highlight three representative individuals tracked across frames, including Track A (ID 6), Track B (ID 9), and Track C (ID 12). The consistent ID labels between the two frames indicate that the proposed tracker maintains identity continuity under dense fish distribution, partial overlap, and visual interference, thereby supporting continuous individual-level body-length monitoring.

Figure 9 shows the tracking effect of the system in complex scenes such as cross-swimming, partial occlusion, and high-density aggregation, and verifies its ability to deal with visual interference and behavioral complexity in a real aquaculture environment, which provides a stable and reliable technical support for continuous and non-contact monitoring of the body length of *Micropterus salmoides*.

The quantitative evaluation results of the improved tracker relative to the initial settings are shown in Table 3. This system performs well on the self-built *Micropterus salmoides* dataset. Compared with the benchmark method DeepSORT, the IDF1 index is increased by 15.6 percentage points, while the body length measurement error is controlled within the range of 5.2 ± 1.8% [26].

### 2.4. Evaluation Index

In order to comprehensively evaluate the performance of the improved algorithm based on YOLOv8 in multi-target tracking tasks, four key evaluation indicators were selected: multi-object tracking accuracy (MOTA), identity F1 score (IDF1), body length measurement error (BLE), and system processing throughput (FPS) [27]. These indicators comprehensively evaluate the tracking system from four aspects: target-matching accuracy, identity consistency, measurement accuracy, and real-time performance.

Multiple object tracking accuracy (MOTA) [28] is one of the core indicators to measure the overall performance of the tracking system, which is mainly used to evaluate the comprehensive ability of the system to detect and track targets in video sequences. MOTA considers three types of error sources: False Positives (FP), False Negatives (FN), and Identity Switches (IDSW) [29]. The calculation method is as shown in Formula (19):

(19) $$\mathrm{MOTA}=1-\frac{\sum_{t}(FP_{t}+FN_{t}+\mathrm{IDS}W_{t})}{\sum_{t}N_{\mathrm{GT},t}}$$

where $FP_{t},\;FN_{t}$ and $IDSW_{t}$ denote the number of false positives, false negatives, and identity switches in frame t, respectively, and $N_{GT,t}$ denote the total number of real targets in frame t. The higher the MOTA value, the more accurate the detection and tracking of the target in the complex scene, and the better the overall tracking performance.

Identification F1 Score (IDF1) is used to measure the ability of the tracking system to maintain the consistency of the target identity between different frames. This indicator takes into account the matching relationship between True Positive ID matches (TP-ID) and predicted/true identities. The calculation method is as shown in Formula (20):

(20) $$\mathrm{IDF}1=\frac{2\cdot \mathrm{TP}-\mathrm{ID}}{2\cdot \mathrm{TP}-\mathrm{ID}+\mathrm{FP}-\mathrm{ID}+\mathrm{FN}-\mathrm{ID}}$$

Among them, TP−ID represents the number of correctly matched identities, FP−ID represents the number of mistakenly matched identities, and FN−ID represents the number of unmatched real identities. The higher the IDF1 value is, the better the system can maintain good identity consistency in complex scenes such as frequent target crossing and occlusion, and has stronger robustness [30].

For the core task of the study, body length monitoring of *Micropterus salmoides*, body length error (BLE) was introduced and used as a special evaluation index to measure the accuracy of the system’s body length estimation during tracking. BLE is defined as the relative error between the predicted body length of the model and the actual body length measured manually. The calculation method is shown in Formula (21):

(21) $${\mathrm{BLE}}_{i}=\left|\frac{l_{\mathrm{pred},i}-l_{\mathrm{gt},i}}{l_{\mathrm{gt},i}}\right|\times 100\%$$

Among them, $l_{pred,i}$ represents the predicted body length of the ith fish, $l_{gt,i}$ represents its true body length. Finally, BLE can be expressed as the average error of all samples.

This index can effectively reflect the accuracy level of the system in non-contact body length measurement, which is of great significance for the evaluation of growth status in aquaculture.

The system processing throughput (FPS) is an important indicator to measure the real-time performance of the tracking system, especially in the runtime assessment under the tested desktop-GPU configuration [31]. The FPS is expressed as the number of video frames that the system can process per second. The calculation method is shown in Formula (22):

(22) $$\mathrm{FPS}=\frac{T}{t_{\mathrm{total}}}$$

Among them, T represents the total number of frames of the test video, $t_{total}$ represents the total time required for the system to process the video. The overall architecture significantly improves the inference throughput of the system through optimization methods such as model compression, layer fusion, and precision quantization.

## 3. Results and Analysis

### 3.1. Training Process and Convergence Analysis

In order to ensure the stability and efficiency of model training, the experiment uses the parameter configuration shown in Table 4 for end-to-end training. A total of 300 training rounds (epochs) were set, the SGD optimizer was used, and YOLOv8s.pt pre-training weights were loaded to accelerate convergence and improve model robustness. The optimal performance checkpoint and the final model weights are automatically saved during the training process [32].

The convergence of the model is evaluated by monitoring the trend of the average accuracy (mAP50) and the loss functions (including the bounding-box regression loss Box_Loss, the distribution focus loss DFL_Loss, and the classification loss Cls_Loss).

From the evolution of the loss function: Box_Loss decreased rapidly from the initial value of 0.7 to 0.3 in the first 100 rounds. After 200 rounds, it entered the fine-tuning stage and further decreased to 0.28. Cls_Loss decreased from 0.8 to 0.3, and the fluctuation range was controlled within ± 0.02 after 250 rounds. DFL_Loss shows the best convergence characteristics, from 0.98 to 0.93, and then stabilized at 0.93 ± 0.005.

All the loss functions have a key turning point between 200 and 250 rounds, and the decline rate is reduced to about 1/5 of the initial stage, indicating that the model gradually enters the convergence state. In the subsequent training (250–300 rounds), the fluctuation of each index is very small: the fluctuation of the loss function is controlled within ± 0.01, the fluctuation of mAP50 is not more than ± 0.2%, and the performance gain is less than 0.3%. These results collectively indicate that the model achieved full convergence at the end of 300 rounds of training and has good generalization ability and optimization integrity.

### 3.2. Contrast Experiment

#### 3.2.1. Performance Comparison of Different Target Detection Models

In order to verify the comprehensive performance advantages of the improved model, we compared a variety of mainstream object detection algorithms on the same test set, including two-stage (Faster R-CNN, Cascade R-CNN), single-stage (SSD, RetinaNet), and YOLO series (YOLOv5-v10) models. The performance comparison is shown in Table 5.

The experimental results show that the model constructed in the study shows significant advantages in the target detection task. Under the lightweight design with only 9.8 MB, 82.7% mAP50, and 81.5% F1-score are achieved, which are 3.2 percentage points higher than the original YOLOv8 and 2.4 percentage points higher than the latest YOLOv10.

In terms of inference throughput, the improved model achieves 88 FPS on an NVIDIA RTX 4060 GPU, which is slightly lower than the 105 FPS of YOLOv10 but significantly better than the two-stage detector (such as Faster R-CNN, only 12 FPS).

In summary, the improved model is in a better position on the accuracy-speed trade-off curve, which provides an efficient and reliable solution for dense small target detection tasks.

#### 3.2.2. Performance Comparison of Different Multi-Target Tracker Combinations

In order to further verify the effectiveness of the proposed tracking system in a complex aquaculture environment, this paper systematically compares and evaluates a variety of mainstream multi-target tracking algorithms (including SORT, DeepSORT, FairMOT, ByteTrack, OC-SORT, BoT-SORT, and the improved method proposed in this paper) under the condition of unified detection input. The evaluation indexes include detection accuracy (mAP50), comprehensive tracking performance (MOTA), identity consistency (IDF1), identity switching times (IDSW), inference throughput, and number of model parameters, so as to comprehensively measure the performance of each method in terms of accuracy, stability, and practicability. The quantitative performance obtained after running the test is shown in Table 6.

The experimental results show that the improved method proposed in this paper shows better performance than the existing technology on multiple key indicators. The mAP50 reaches 82.7%, which is 1.5 percentage points higher than that of the sub-optimal BoT-SORT, indicating that it has a stronger ability in target positioning and detection reliability. The IDF1 index is increased to 81.5%, which is 3.2 percentage points higher than that of BoT-SORT. At the same time, IDSW is reduced to 39 times, which is 48% lower than that of FairMOT, which significantly alleviates the problem of identity switching in high-density fish swarm scenarios and reflects better trajectory continuity and identity retention ability. In terms of efficiency, the system inference throughput is maintained at 88 FPS, which meets the real-time monitoring requirements, and the number of model parameters is only 9.8 MB, which is significantly lower than that of DeepSORT (58.7 MB) and OC-SORT (36.8 MB), showing better lightweight characteristics.

### 3.3. Ablation Experimental Analysis

#### 3.3.1. Preliminary Validations

Before presenting the main ablation analysis of the proposed detection and tracking modules (Section 3.3.2), we first report two preliminary validation experiments that underpin the methodological choices described in Section 2: the efficiency and accuracy of the collaborative labeling and data augmentation workflow, and the sensitivity of the geometric measurement pipeline to depth estimation error. These results justify the design decisions but are distinct from the core ablation of the proposed framework; they are therefore presented separately here to maintain a clear separation between preliminary validations and the primary experimental evaluation.

Collaborative labeling and data augmentation. The automated pre-labeling stage achieved satisfactory baseline accuracy under clear water conditions, with 71.3% of initial bounding boxes exceeding an IoU of 0.85 against manual expert annotations. The collaborative labeling strategy (automated pre-detection followed by expert review) reduced total annotation time from an estimated 126 h (fully manual mode) to 42 h, a 66.7% efficiency improvement. On the data side, the combined geometric and color augmentation strategy raised mAP50 from 82.3% to 89.5%—a 7.2 percentage point improvement—while increasing per-frame inference time only marginally (from 18.2 ms to 21.4 ms) and parameter count from 3.2 M to 3.3 M. These complementary results confirm that both efficient annotation workflows and data-level enhancements provide substantial practical gains at negligible computational cost, validating the methodological investments described in Section 2.1.

Depth sensitivity analysis. Because the geometric measurement pipeline assumes a nominal fish swimming depth of h = 0.8 m (Section 2.2.2), we evaluated the impact of depth estimation error on body length accuracy by varying h across the range [0.4 m, 1.2 m]. The results (Table 7) show that BLE remains within acceptable limits (5.2–6.8%) across the entire tested range. Deviation from the nominal depth by ±0.2 m produces only modest error increases (5.5% at h = 1.0 m; 5.8% at h = 0.6 m), while a ±0.4 m deviation raises BLE to 6.3% (h = 1.2 m) and 6.8% (h = 0.4 m), respectively. The absolute error for a representative 25 cm fish stays below 1.7 cm in all scenarios. These findings indicate that the measurement pipeline is moderately sensitive to depth estimation; the ±0.2 m deviation expected under typical cruising behavior (Section 2.2.2) falls within the tolerance range for aquaculture management (5–10%), but future work should incorporate per-fish depth estimation (e.g., stereo vision or learned depth regression) to further reduce this error source.

#### 3.3.2. Ablation Experimental Validation and Analysis

In order to systematically evaluate the contribution of each improved module, we conducted detailed ablation experiments. The specific quantitative results of the ablation experiment are shown in Table 8. Based on the original YOLOv8s model, various improvements are gradually introduced, and the performance changes are recorded.

To evaluate the contribution of each module toward the ultimate application goal of body length estimation, the body length error (BLE) was measured for each incremental configuration on the same 15-fish validation set. The results show a progressive reduction in BLE from 8.5 ± 2.3% (baseline) to 5.2 ± 1.8% (full model), with the largest single improvement contributed by the ReID branch (ΔBLE = −0.7%). Based on the baseline model, a shallow feature fusion mechanism (FPN-Aug) is introduced. By enhancing the high-resolution feature path of P2 level (1/4 input resolution) in the feature pyramid network, the perception ability of long-distance or small-scale fish is significantly improved. The improvement increases mAP50-95 by 1.3 percentage points and reduces the number of missed detections (FN) by 22 cases, which verifies the effectiveness of multi-scale detail information fusion for small-target detection. Critically, this detection improvement translates into a BLE reduction from 8.5% to 8.0%, demonstrating that enhanced small-target perception directly benefits length measurement accuracy. The bounding box regression process is further optimized, and the improved distributed focus loss (DFL) is used to enhance the positioning accuracy. The mAP50-95 is further improved to 67.1%, and the number of false positives (FP) is significantly reduced to 97, indicating that the optimization of the regression mechanism helps to suppress redundant prediction. The corresponding BLE improvement from 8.0% to 7.2% confirms that more accurate bounding box localization propagates into reduced length estimation error.

After integrating all the improvements, the final complete model achieves mAP50-95 of 69.1% while maintaining real-time throughput of 88 FPS, and the number of false positives (FP) and false negatives (FN) are reduced to 58 and 112, respectively, which is 53.2% and 40.1% better than the baseline model. The corresponding BLE is reduced to 5.2 ± 1.8%, representing a 38.8% improvement over the baseline and meeting the 5–10% tolerance requirement for aquaculture growth monitoring. These results demonstrate that incremental gains in detection and tracking metrics propagate into practically meaningful reductions in body length estimation error.

The depth test in typical complex scenes further verifies the adaptability of the model. In turbid water with turbidity greater than 50 NTU, mAP50 increased from 71.2% to 79.1%, a relative increase of 7.9%, indicating that the enhanced feature expression and data enhancement strategy effectively improved the robustness to low-quality images. In the high-density interaction scene where fish frequently cross, IDSW is greatly reduced from 89 times to 27 times, and the trajectory continuity is significantly enhanced. The corresponding BLE under high-density conditions improved from 9.8 ± 2.5% (baseline) to 6.1 ± 2.0% (full model), confirming that reduced identity switching directly stabilizes body length time-series estimates. In the face of motion blur caused by fast motion, the missed detection rate is reduced by 41.3% (FN from 187 to 112), which reflects the adaptability of detection and tracking collaborative optimization to a dynamic environment.

The above results systematically verify the rationality and effectiveness of each module design, and fully prove that the proposed method has good robustness, stability, and engineering application potential in a real aquaculture environment.

### 3.4. Application-Level Validation: Body Length Error Analysis

While the preceding sections evaluate detection and tracking performance through component-level metrics (mAP, MOTA, IDF1), the ultimate criterion for the proposed framework is its impact on body length estimation accuracy. This section explicitly connects component-level improvements to application-level body length error (BLE) reduction.

Propagating detection improvements to BLE. Ablation experiments (Section 3.3) revealed that the FPN-Aug shallow feature fusion mechanism improved mAP50 from 79.5% to 80.8%, with corresponding BLE reduction from 8.5% to 8.0%. The subsequent DFL regression optimization raised mAP50 to 81.2% and further reduced BLE to 7.2%. These results confirm a direct relationship between enhanced small-target perception and improved length measurement accuracy.

Propagating tracking improvements to BLE. The integration of the MobileNetV3_ReID module and the hybrid matching strategy yielded the most substantial BLE improvement. While detection gains (FPN-Aug + DFL) reduced BLE by 1.3 percentage points, tracking improvements (ReID + Hybrid-Match) contributed an additional 2.0 percentage points of BLE reduction (from 7.2% to 5.2%). This disproportionate contribution confirms that tracking stability—specifically, reduced identity switching (IDSW decreased from 89 to 39)—is critical for reliable body length time-series estimation.

Comparison with baseline trackers. Table 6 provides complementary evidence: when the proposed detector was paired with DeepSORT, BLE remained high at 8.7 ± 2.5%, despite reasonable MOTA (68.7%). Substituting the proposed tracking system reduced BLE to 5.2 ± 1.8%—a 40.2% improvement. This demonstrates that both detection and tracking components must be jointly optimized to achieve application-relevant accuracy.

Practical significance. The final BLE of 5.2 ± 1.8% (MAE = 1.35 cm) falls comfortably within the 5–10% tolerance range accepted for aquaculture management decisions. The Pearson correlation between automated and manual measurements (r = 0.972, *p* < 0.001) confirms that the system captures true biological variation rather than noise. Critically, the 38.8% BLE improvement over the baseline (8.5% to 5.2%) was achieved without sacrificing throughput (88 FPS), confirming that the integrated framework delivers both application-level accuracy and real-time feasibility.

## 4. Discussion

### 4.1. Application-Level Contribution of Body-Length Estimation

The central contribution of this study is the demonstration that a tightly integrated detection-tracking-measurement pipeline can achieve body-length estimation accuracy sufficient for production aquaculture management. The system achieved a body-length error (BLE) of 5.2 +/− 1.8% (MAE = 1.35 cm, RMSE = 1.58 cm) relative to manual caliper measurements, with a Pearson correlation coefficient of r = 0.972 across 15 validation fish ranging from 18.5 to 32.7 cm in total length. This error level falls comfortably within the 5-10% tolerance range accepted for aquaculture growth-monitoring decisions, where body length is used to determine feeding ratios, stocking density adjustments, and harvest timing. For fish in the 20–30 cm range typical of grow-out ponds, a 5.2% error corresponds to approximately 1.0–1.6 cm of absolute uncertainty, acceptable for most management protocols. The high correlation (r = 0.972) confirms that the system captures true biological variation in body length rather than random measurement noise, establishing the practical utility of the proposed framework for longitudinal growth analysis.

From a practical perspective, the integration of these three functional modules addresses a critical gap in existing aquaculture technology. Most prior studies have focused on isolated tasks—either the detection of fish targets in single frames or the tracking of swimming trajectories without morphological measurement. For instance, Mei et al. [33] reported a 1.0% improvement in recall by enhancing shallow feature propagation for small fish detection, yet their framework did not incorporate tracking or length estimation. Similarly, Jiang et al. [34] achieved 80.10% MOTA in poultry tracking using BoT-SORT, but their system was designed for terrestrial environments without geometric measurement capabilities. By unifying detection, tracking, and length estimation within a single framework, this study enables continuous, non-invasive monitoring of individual growth trajectories over time, a capability that is essential for precision aquaculture management but has been largely absent from existing solutions.

### 4.2. Why Detection and Tracking Jointly Reduce Body-Length Error

Ablation experiments revealed that both detection and tracking modules contribute to BLE reduction, but through different mechanisms and with unequal magnitudes. The detection enhancements-FPN-Aug shallow fusion, and DFL regression-together reduced BLE by 1.3 percentage points (from 8.5% to 7.2%). This contribution operates primarily through improved localization accuracy: FPN-Aug enhances small-target perception by preserving high-resolution features at the P2 level (1/4 input resolution), reducing missed detections of distant or partially occluded fish, while DFL models the distribution of bounding box boundaries to capture uncertainty in fish boundary localization caused by flexible body postures [33,35]. In contrast, the tracking improvements—ReID feature extraction and hybrid matching strategy—together reduced BLE by 2.0 percentage points (from 7.2% to 5.2%), exceeding the detection contribution. This larger tracking effect arises because body length estimation relies on temporal continuity: when identity switches fragment trajectories, the weighted moving average (WMA) smoothing cannot operate on continuous sequences, producing noisy length estimates. The hybrid matching strategy reduces ID switches from 89 (baseline) to 39 by integrating spatial overlap (IoU), appearance cosine similarity, and motion consistency within a unified cost matrix, ensuring that each tracked identity yields a stable body-length time series [34,36]. These results establish that both detection and tracking must be jointly optimized to achieve application-relevant BLE accuracy; improvements in either component alone are insufficient.

### 4.3. Limitations and Future Work

Several limitations of this study should be acknowledged. The experimental dataset was collected from a single aquaculture pond over a 24 h period. Although this controlled setting ensured consistency in water quality, lighting, and camera configuration, it limits the assessment of model generalization across different pond geometries, camera angles, and environmental conditions. Cross-tank validation is necessary to determine whether the detection and tracking modules maintain performance when transferred to facilities with different stocking densities, turbidity levels, or infrastructure layouts [37]. Generalization to different tanks, seasons, or operational conditions remains to be validated in future work.

The body length validation experiment involved 15 individuals randomly captured from the pond. While the correlation with manual measurements was strong (r = 0.972), the relatively small sample size restricts the statistical confidence in the error estimates, particularly for size classes that were underrepresented in the sampled population. Future validation should encompass a larger and more diverse sample spanning the full range of body lengths present in production ponds.

Although the system specifications (9.8 MB, 18.5 GFLOPs) suggest potential for edge deployment, no actual edge device testing was conducted in this study. The inference speed of 88 FPS was measured on desktop GPU hardware, and the performance characteristics on embedded platforms such as Jetson AGX Xavier or Jetson Nano remain to be determined. The accuracy degradation and latency implications of model quantization and TensorRT optimization also require systematic evaluation.

The current framework focuses exclusively on body length as a morphological indicator. While body length is a primary metric for growth assessment, weight estimation, condition factor analysis, and health status evaluation would provide a more comprehensive picture of population status. Extension to these additional indicators would require supplementary training data and potentially architectural modifications to the measurement module.

Future research directions include the following: (1) introducing temporal encoding strategies, such as transformer-based architectures, to enhance detection and tracking robustness by leveraging multi-frame context information [38]; (2) conducting cross-tank and cross-species validation to establish the generalization boundaries of the proposed framework; (3) integrating weight estimation through allometric modeling or direct regression from appearance features; and (4) deploying and benchmarking the system on representative edge computing devices to validate real-world feasibility and identify further optimization opportunities.

## 5. Conclusions

This study proposes an integrated multi-target detection and tracking framework based on improved YOLOv8s for real-time, non-contact monitoring of *Micropterus salmoides* body length in intensive recirculating aquaculture systems. The main contributions are

(1) A shallow feature fusion mechanism (FPN-Aug) at the P2 level that enhances the network’s perception of small fish targets, combined with a Body Shape-aware Loss incorporating auxiliary keypoint supervision for anatomically consistent bounding-box regression. These improvements raised mAP50 from 79.5% to 82.7% and mAP50-95 from 65.2% to 69.1%.

(2) A lightweight MobileNetV3_ReID module adapted for fish appearance feature extraction, achieving 74.6% mAP and 87.3% Rank-1 accuracy on the FishReID benchmark with 75% lower feature dimension and 65.5% lower inference time than ResNet50.

(3) A hybrid data association strategy integrating spatial overlap (IoU), appearance feature similarity, and motion consistency within a dynamic-threshold Hungarian framework with EMA feature update. This reduced identity switches from 89 (baseline) to 39 (56.2% decrease) and achieved 81.5% IDF1, outperforming the strongest baseline BoT-SORT by 3.2 percentage points.

The system maintains real-time throughput of 88 FPS on desktop GPU hardware with 9.8 MB, and controls relative body-length error at 5.2 ± 1.8% (approximately 1.3–1.6 cm absolute error for typical 25–30 cm fish), representing a 38.8% improvement over the baseline. Application-level validation (Section 3.4) further demonstrates that these component-level gains are not merely incremental metric improvements: detection enhancements (FPN-Aug + DFL) reduced BLE by 1.3 percentage points by improving small-target localization, while tracking optimizations (ReID + Hybrid-Match) contributed an additional 2.0 percentage points by stabilizing identity consistency—confirming that both detection and tracking must be jointly optimized to achieve practically relevant body length estimation accuracy. The compact model design suggests potential for future edge deployment, though embedded hardware validation remains to be conducted.

This work provides a technically feasible path for individual growth tracking in aquaculture. Future directions include formal temporal encoding (e.g., transformer or Mamba-based sequence modeling), knowledge distillation for further lightweighting, three-dimensional pose estimation fusion, cross-tank validation, and integration with multi-modal sensor systems to advance the transition from experience-based to data-driven aquaculture management.

## Figures and Tables

**Table 1:** Summary of environmental conditions in the original dataset and synthetically generated through augmentation.

Condition	Present in Original Data	Synthetically Augmented	Method
Variable fish density (15–35 fish/frame)	Yes	—	Natural variation
Diurnal illumination variation	Yes	—	24 h collection period
Moderate turbidity (20–40 NTU)	Yes	—	Natural water conditions
Extreme turbidity (>50 NTU)	Partial	Yes	Algae suspension noise (density ≤ 15%)
Severe occlusion/crossing	Partial	Yes	Random cropping + Mosaic splicing
Water refraction distortion	Yes	Yes	Elastic deformation
Spectral color shift	Yes	Yes	Blue channel gain (+15%), red channel suppression (−10%)
Cross-device illumination	No	Yes	CycleGAN style transfer
Sensor noise	No	Yes	Lightweight anti-disturbance generation module

**Table 2:** FishReID benchmark test set evaluation results.

Model	Feature Dimension	Inference Time (ms)	mAP (%)	Rank-1 (%)
ResNet50	2048	28.4	76.3	88.5
MobileNetV3 (original)	960	12.7	68.2	82.1
Ours (improved)	512	9.8	74.6	87.3

Note: All models are tested on an RTX 4060 GPU with batch size 1. The proposed method achieves a favorable trade-off between model compactness, speed, and accuracy, reducing feature dimension by 75% and inference time by 65.5% compared to ResNet50, while maintaining competitive retrieval and detection performance.

**Table 3:** Tracking performance comparison under controlled detection input.

Evaluation Metric	Ours	DeepSORT	Improvement	Measurement Condition
MOTA (%)	82.3 ± 2.1	68.7 ± 3.4	+13.6	Fish density: 15–20 individuals per frame
IDF1 (%)	85.1 ± 1.8	73.5 ± 2.7	+11.6	Video resolution: 1920 × 1080
FP (count/frame)	1.2 ± 0.3	2.8 ± 0.6	−57.1%	Detection confidence threshold: 0.5
FN (count/frame)	0.8 ± 0.2	1.5 ± 0.4	−46.7%	Water turbidity: 30–50 NTU
IDSW (count/k-frame)	9.7 ± 2.4	15.3 ± 3.1	−36.6%	Tracking duration ≥ 5 min
Body length error (%)	5.2 ± 1.8	8.7 ± 2.5	−40.2%	Calibrated with reference board
Processing throughput (FPS)	28.4	35.2	−19.3%	RTX 4060 GPU
Max tracking capacity	52	38	+36.8%	Critical density with MOTA > 75%

Note: Values are mean ± standard deviation unless otherwise specified. To isolate the tracker evaluation from the detector, all results in this table were obtained using the unmodified baseline detection model. MOTA: multi-object tracking accuracy; IDF1: identity F1 score; FP: false positives; FN: false negatives; IDSW: ID switches. The proposed system achieves significant improvements in tracking accuracy and robustness, with a moderate trade-off in processing throughput.

**Table 4:** The main training parameters of model construction.

Parameter	Value
Epochs	300
Patience (early stopping)	30
Batch size	8
Input size	640 × 640
Optimizer	SGD
Initial learning rate	0.01
Learning rate scheduler	Cosine annealing
Pre-trained weights	YOLOv8s.pt
Dataset split	Train:Validation:Test = 7:2:1

Note: All experiments are conducted using the Ultralytics YOLO framework. The model is trained for up to 300 epochs, with early stopping applied if no improvement in validation mAP@0.5:0.95 is observed for 30 consecutive epochs.

**Table 5:** Performance comparison of different detection models.

Model	Backbone	Parameters (MB)	F1-Score (%)	Precision (%)	Recall (%)	mAP50 (%)	Throughput (FPS)
Faster R-CNN	ResNet50	41.2	68.3	72.1	64.8	69.5	12
Cascade R-CNN	ResNet101	69.7	71.5	75.2	68.1	72.8	9
SSD	VGG16	26.8	65.2	70.3	60.7	63.4	35
RetinaNet	ResNet50-FPN	37.7	67.8	71.5	64.3	68.9	18
YOLOv5	CSPDarknet53	7.5	74.6	76.8	72.5	75.2	95
YOLOv6	EfficientRep	8.7	75.1	77.3	73.0	76.0	98
YOLOv7	ELANNet	37.2	76.8	78.5	75.2	77.9	85
YOLOv8	CSPDarknet53	11.4	78.3	80.1	76.6	79.5	92
YOLOv10	CSPDarknet53	8.2	79.2	81.0	77.5	80.3	105
Ours	CSPDarknet	9.8	81.5	83.2	79.8	82.7	88

Note: All models were evaluated on the same test set with identical input resolution (640 × 640). YOLO-series models use small-weight variants for fair comparison. Speed measured on NVIDIA RTX 4060 GPU.

**Table 6:** Performance comparison of different tracker schemes.

Algorithm	Backbone Network	mAP50 (%)	MOTA (%)	IDF1 (%)	IDSW	Speed (FPS)	Params (MB)	BLE (%)
SORT	YOLOv3	68.2	62.1	65.3	142	120	61.5	11.8 ± 3.2
DeepSORT	YOLOv5 + ResNet50	75.4	68.7	72.5	89	45	58.7	8.7 ± 2.5
FairMOT	DLA-34	77.1	71.2	74.8	75	38	27.3	8.0 ± 2.4
ByteTrack	YOLOX-s	79.3	73.5	76.9	63	65	8.9	7.5 ± 2.2
OC-SORT	YOLOv7	80.1	74.8	77.5	51	52	36.8	6.9 ± 2.0
BoT-SORT	YOLOv8	81.2	75.6	78.3	47	60	11.4	6.2 ± 1.9
Ours	YOLOv8s + MobileNetV3	82.7	76.9	81.5	39	88	9.8	5.2 ± 1.8

Note: mAP50 is identical across all trackers because the same improved YOLOv8s detector output is used as input. mAP50 differences between Table 5 and Table 6 reflect detector evaluation vs. tracker evaluation protocols. MOTA: multi-object tracking accuracy; IDF1: identity F1 score; IDSW: number of identity switches; FPS: frames per second; Params: memory footprint. BLE: body length error, measured as mean relative error (%) against manual caliper measurements on a 15-fish validation set. Tracker comparison conducted on NVIDIA RTX 4060 GPU.

**Table 7:** Sensitivity of body length measurement error to fish depth estimation error.

Assumed Depth h (m)	Deviation from Nominal (m)	Body Length Error Rate(%)	Absolute Error (cm, for 25 cm Fish)
0.4	−0.4	6.8	1.70
0.6	−0.2	5.8	1.45
0.8	0	5.2	1.30
1.0	+0.2	5.5	1.38
1.2	+0.4	6.3	1.58

Note: Error values represent the mean relative error across 15 manually measured validation fish. The analysis indicates that the measurement pipeline is moderately sensitive to depth estimation error, with errors increasing by approximately 1.0–1.6 percentage points per 0.4 m deviation from the nominal depth. Future work may incorporate per-fish depth estimation from stereo vision or learned depth regression to reduce this source of error.

**Table 8:** Comparison of ablation experimental results.

Metric	Item	Baseline	+FPN-Aug	+DFL	+ReID	+Hybrid-Match	Full Model
Modules Enabled	FPN-Augmentation	×	√	√	√	√	√
	DFL	×	×	√	√	√	√
	ReID Branch	×	×	×	√	√	√
	HMS	×	×	×	×	√	√
Performance	mAP50 (%)	79.5	80.8	81.2	81.7	82.3	82.7
	mAP50-95 (%)	65.2	66.5	67.1	67.8	68.5	69.1
	IDF1 (%)	76.2	77.5	78.1	79.8	80.5	81.5
	MOTA (%)	72.8	74.1	74.9	75.6	76.3	76.9
	IDSW	89	82	75	63	52	39
	FP (count/frame)	124	108	97	85	72	58
	FN (count/frame)	187	165	152	138	125	112
	FPS	95	92	90	85	83	88
	Parameters (MB)	7.5	8.1	8.1	9.8	9.8	9.8
	FLOPs (G)	16.3	17.8	17.8	18.5	18.5	18.5
	BLE(%)	8.5±2.3	8.0	7.2	6.5	5.8	5.2

Note: ×: module disabled; √: module enabled. Improvements over the previous row are shown in parentheses. DFL: distribution focal loss; HMS: hybrid matching strategy; mAP50: mean average precision at IoU = 0.5; mAP50-95: mAP averaged over IoU thresholds from 0.5 to 0.95; IDF1/MOTA: tracking accuracy metrics; IDSW: ID switches; FP: false positives; FN: false negatives; FPS: frames per second; Params: memory footprint; FLOPs: floating point operations. BLE: body length error, measured as mean relative error (%) against manual caliper measurements on a 15-fish validation set.

## Data Availability

The datasets generated and/or analyzed during the current study are not publicly available due to institutional restrictions, but may be available from the corresponding author upon reasonable request.

## References

[1] Shafait F., Harvey E.S., Shortis M.R., Mian A., Ravanbakhsh M., Seager J.W., Culverhouse P.F., Cline D.E., Edgington D.R. (2017). Towards Automating Underwater Measurement of Fish Length: A Comparison of Semi-Automatic and Manual Stereo–Video Measurements. ICES J. Mar. Sci..

[2] Navarro A., Lee-Montero I., Santana D., Henríquez P., Ferrer M.A., Morales A., Soula M., Badilla R., Negrín-Báez D., Zamorano M.J. (2016). IMAFISH_ML: A Fully-Automated Image Analysis Software for Assessing Fish Morphometric Traits on Gilthead Seabream (*Sparus aurata* L.), Meagre (*Argyrosomus regius*) and Red Porgy (*Pagrus pagrus*). Comput. Electron. Agric..

[3] Salman A., Jalal A., Shafait F., Mian A., Shortis M., Seager J., Harvey E. (2016). Fish Species Classification in Unconstrained Underwater Environments Based on Deep Learning. Limnol. Oceanogr. Meth..

[4] Al-Jubouri Q., Al-Nuaimy W., Al-Taee M., Young I. (2017). An Automated Vision System for Measurement of Zebrafish Length Using Low-Cost Orthogonal Web Cameras. Aquac. Eng..

[5] Huang K., Li Y., Suo F., Xiang J. (2020). Stereo vison and mask-RCNN segmentation based 3D points cloud matching for fish dimension measurement. Proceedings of the 2020 39th Chinese Control Conference (CCC), Shenyang, China, 27–29 July 2020.

[6] Ling S., Hong X., Liu Y. (2024). YOLO-APDM: Improved YOLOv8 for Road Target Detection in Infrared Images. Sensors.

[7] Fernandes A.F.A., Turra E.M., de Alvarenga É.R., Passafaro T.L., Lopes F.B., Alves G.F.O., Singh V., Rosa G.J.M. (2020). Deep Learning Image Segmentation for Extraction of Fish Body Measurements and Prediction of Body Weight and Carcass Traits in Nile Tilapia. Comput. Electron. Agric..

[8] Feng D., Liu H., Wu L., Ying X., Qu X., Gao Y., Gui F., Zhou C. (2025). Morphological Feature Assessment of *Larimichthys crocea* for Quality Grading: A Computer Vision Approach. Aquac. Int..

[9] Cong X., Tian Y., Quan J., Qin H., Li Q., Li R. (2024). Machine Vision-Based Estimation of Body Size and Weight of Pearl Gentian Grouper. Aquac. Int..

[10] Zhao H., Wu Y., Qu K., Cui Z., Zhu J., Li H., Cui H. (2024). Vision-Based Dual Network Using Spatial-Temporal Geometric Features for Effective Resolution of Fish Behavior Recognition with Fish Overlap. Aquac. Eng..

[11] Hua J., He R., Zeng Y., Chen Q. (2025). HDMS-YOLO: A Multi-Scale Weed Detection Model for Complex Farmland Environments. Front. Plant Sci..

[12] She X., Tang Z., Pan X., Zhao J., Liu W. (2025). DBFormer: A Dual-Branch Adaptive Remote Sensing Image Resolution Fine-Grained Weed Segmentation Network. Remote Sens..

[13] Zhang Z. (2000). A Flexible New Technique for Camera Calibration. IEEE Trans. Pattern Anal. Mach. Intell..

[14] Ran X., Li B., Zhang Y., Kong M., Duan Q. (2024). Anomalous White Shrimp Detection in Intensive Farming Based on Improved YOLOv8. Aquac. Eng..

[15] Zhang Z., Li J., Su C., Wang Z., Li Y., Li D., Chen Y., Liu C. (2024). A Method for Counting Fish Based on Improved YOLOv8. Aquac. Eng..

[16] Zhang Y., Hu Z., Liu J., Li Y., Lin J., Wang Y., Yu H. (2025). PUFFER-DETR: Tiger Puffer Similar Abnormal Behavior Recognition Based on Transformer. Aquac. Eng..

[17] Liu C., Wang Z., Li Y., Zhang Z., Li J., Xu C., Du R., Li D., Duan Q. (2023). Research Progress of Computer Vision Technology in Abnormal Fish Detection. Aquac. Eng..

[18] Wang Z., Qian R., Deng H., Zhou L., Ling J. (2025). Precise Feeding Technology for Outdoor Pond Aquaculture Based on Detection and Counting Method. Aquac. Eng..

[19] Liu H., Cui M., Gu H., Feng J., Zeng L. (2025). A Fast and Dynamic Tracking-Based Micropterus Salmoides Fry Counting Method in Highly Occluded Scenarios. Aquac. Eng..

[20] Zhao Y., Chen M., Feng G., Zhai W., Xiao P., Huang Y., Zhu J. (2025). MC-MobileFishNet: An Efficient Algorithm for Phenotypic Analysis in Heat-Resistant Breeding of Micropterus Salmoides Using Keypoint Detection. Aquac. Eng..

[21] Gu Z., He D., Huang J., Chen J., Wu X., Huang B., Dong T., Yang Q., Li H. (2024). Simultaneous Detection of Fruits and Fruiting Stems in Mango Using Improved YOLOv8 Model Deployed by Edge Device. Comput. Electron. Agric..

[22] Nie L., Li B., Jiao F., Lu W., Shi X., Song X., Shi Z., Yang T., Du Y., Liu Z. (2024). EVIT-YOLOv8: Construction and Research on African Swine Fever Facial Expression Recognition. Comput. Electron. Agric..

[23] Xie Z., Liu W., Li Y., Du J., Long T., Xu H., Long Y., Zhao J. (2025). Enhanced Litchi Fruit Detection and Segmentation Method Integrating Hyperspectral Reconstruction and YOLOv8. Comput. Electron. Agric..

[24] Yu J., Wang G., Li X., Du Z., Xu W., Akhter M., Li D. (2025). SDYOLO-Tracker: An Efficient Multi-Fish Hypoxic Behavior Recognition and Tracking Method. Comput. Electron. Agric..

[25] Li Y., Tan H., Deng Y., Zhou D., Zhu M. (2025). Hypoxia Monitoring of Fish in Intensive Aquaculture Based on Underwater Multi-Target Tracking. Comput. Electron. Agric..

[26] Su R., Yue J., Li Z., Jia S., Sheng G. (2024). Detection and Counting Method of Juvenile Abalones Based on Improved SSD Network. Inf. Process. Agric..

[27] Xu J., Yang S., Liang Q., Zheng Z., Ren L., Fu H., Yang P., Xie W., Yang D. (2025). Transillumination Imaging for Detection of Stress Cracks in Maize Kernels Using Modified YOLOv8 after Pruning and Knowledge Distillation. Comput. Electron. Agric..

[28] Sulzbach E., Scheeren I., Torres Veras M.S., Tosin M.C., Ellert Kroth W.A., Merotto A., Markus C. (2025). Deep Learning Model Optimization Methods and Performance Evaluation of YOLOv8 for Enhanced Weed Detection in Soybeans. Comput. Electron. Agric..

[29] Yin Y., Sun X., Yu G., Wang J., Li D., Wang Y. (2025). CBFW-YOLOv8: Automated Recognition Method for Fish Body Surface Diseases in Recirculating Aquaculture Systems. Comput. Electron. Agric..

[30] Zhang H., Li W., Qi Y., Liu H., Li Z. (2023). Dynamic Fry Counting Based on Multi-Object Tracking and One-Stage Detection. Comput. Electron. Agric..

[31] Huang Z., Zhao H., Cui Z., Wang L., Li H., Qu K., Cui H. (2024). Early Warning System for Nocardiosis in Largemouth Bass (*Micropterus salmoides*) Based on Multimodal Information Fusion. Comput. Electron. Agric..

[32] Xu W., Liu C., Wang G., Zhao Y., Yu J., Muhammad A., Li D. (2024). Behavioral Response of Fish under Ammonia Nitrogen Stress Based on Machine Vision. Eng. Appl. Artif. Intell..

[33] Mei Y., Chen Y., Liu Y., Yu H., Yang L., Li D. (2026). MFSD-YOLO: A Multi-Scenario Fish Small Target Detection Method in Aquaculture. Aquac. Eng..

[34] Jiang D., Wang H., Li T., Gouda M.A., Zhou B. (2025). Real-Time Tracker of Chicken for Poultry Based on Attention Mechanism-Enhanced YOLO-Chicken Algorithm. Comput. Electron. Agric..

[35] Zhu Y., Zhao Y., He Y., Wu B., Su X. (2025). YOLO-WildASM: An Object Detection Algorithm for Protected Wildlife. Animals.

[36] Li Z., Cheng G., Yang L., Han S., Wang Y., Dai X., Fang J., Wu J. (2025). Method for Dairy Cow Target Detection and Tracking Based on Lightweight YOLO v11. Animals.

[37] Wu A., Li K., Song Z., Lou X., Hu P., Yang W., Wang R. (2025). Deep Learning for Sustainable Aquaculture: Opportunities and Challenges. Sustainability.

[38] Yao M., Huo Y., Tian Q., Zhao J., Liu X., Wang R., Xue L., Wang H. (2025). FMRFT: Fusion Mamba and DETR for Query Time Sequence Intersection Fish Tracking. Comput. Electron. Agric..

